# Genome-Wide Identification and Expression Analysis of Heat Shock Protein 70 (*HSP70*) Gene Family in Pumpkin (*Cucurbita moschata*) Rootstock under Drought Stress Suggested the Potential Role of these Chaperones in Stress Tolerance

**DOI:** 10.3390/ijms23031918

**Published:** 2022-02-08

**Authors:** Marzieh Davoudi, Jinfeng Chen, Qunfeng Lou

**Affiliations:** State Key Laboratory of Crop Genetics and Germplasm Enhancement, College of Horticulture, Nanjing Agricultural University, Nanjing 210095, China; 2018204052@njau.edu.cn (M.D.); jfchen@njau.edu.cn (J.C.)

**Keywords:** heat shock proteins, chaperones, drought stress, pumpkin, phylogeny, expression pattern

## Abstract

Heat shock protein 70s (HSP70s) are highly conserved proteins that are involved in stress responses. These chaperones play pivotal roles in protein folding, removing the extra amounts of oxidized proteins, preventing protein denaturation, and improving the antioxidant system activities. This conserved family has been characterized in several crops under drought stress conditions. However, there is no study on *HSP70s* in pumpkin (*Cucurbita moschata*). Therefore, we performed a comprehensive analysis of this gene family, including phylogenetic relationship, motif and gene structure analysis, gene duplication, collinearity, and promoter analysis. In this research, we found 21 *HSP70s* that were classified into five groups (from A to E). These genes were mostly localized in the cytoplasm, chloroplast, mitochondria, nucleus, and endoplasmic reticulum (ER). We could observe more similarity in closely linked subfamilies in terms of motifs, the number of introns/exons, and the corresponding cellular compartments. According to the collinearity analysis, gene duplication had occurred as a result of purifying selection. The results showed that the occurrence of gene duplication for all nine gene pairs was due to segmental duplication (SD). Synteny analysis revealed a closer relationship between pumpkin and cucumber than pumpkin and Arabidopsis. Promoter analysis showed the presence of various cis-regulatory elements in the up-stream region of the *HSP70* genes, such as hormones and stress-responsive elements, indicating a potential role of this gene family in stress tolerance. We furtherly performed the gene expression analysis of the *HSP70s* in pumpkin under progressive drought stress. Pumpkin is widely used as a rootstock to improve stress tolerance, as well as fruit quality of cucumber scion. Since stress-responsive mobile molecules translocate through vascular tissue from roots to the whole plant body, we used the xylem of grafted materials to study the expression patterns of the HSP70 (potentially mobile) gene family. The results indicated that all *CmoHSP70s* had very low expression levels at 4 days after stress (DAS). However, the genes showed different expression patterns by progressing he drought period. For example, the expression of *CmoHSP70*-4 (in subgroup E) and *CmoHSP70*-14 (in subgroup C) sharply increased at 6 and 11 DAS, respectively. However, the expression of all genes belonging to subgroup A did not change significantly in response to drought stress. These findings indicated the diverse roles of this gene family under drought stress and provided valuable information for further investigation on the function of this gene family, especially under stressful conditions.

## 1. Introduction

Environmental threats are becoming more serious because of climate change and global warming [1]. Plants, as the organisms that are not able to move, have mechanisms to survive or adapt to stressful conditions [2]. For instance, evolutionary analysis has shown that plants have more stress-responsive genes, such as heat shock proteins (*HSPs*), than other organisms as a result of whole-genome duplications to cope with adverse conditions [3]. The *HSP* superfamily, which is highly conserved among organisms [4], is divided into different families based on their molecular weight, including *HSP100*, *HSP90*, *HSP70*, *HSP60*, and small *HSPs* [5]. Among chaperones, *HSP70s*, which are highly conserved in prokaryotes (*DnaK*) and eukaryotes (*HSP70*) [6,7], play dominant roles in plant development. These chaperones also have various functions, such as assisting proteins in correct folding, protecting proteins against misfolding [8], repairing the damaged proteins, and removing the extra amounts of damaged proteins to avoid oxidative stress [9]. There are three conserved domains in this gene family, including C-terminal substrate binding domain, or SBD (about 10 kDa for biding to the substrate), intermediate domain (about 15 kDa), and N-terminal nucleotide binding domain, or NBD (44 kDa for ATP-binding) [10].

One of the critical phenomena that determines the function of molecular chaperones is post-translational modifications (PTMs), such as phosphorylation, ubiquitination, AMPylation, oxidation, and so on [11]. These modifications and their effects on the function of chaperones are also considered as “chaperone code” [12]. As an example, phosphorylation of HSP70 at SBD and NBD regions improves the binding affinity of this chaperone to its targets [13]. SBD and NBD are two functionally crucial regions for PTM, since the mutation in these sites represses the ability of HSP70 to repair misfolded proteins [14]. Ubiquitination of HSP70 influences proteasomal degradation. AMPylation is another PTM manner that regulates the amount of activated/inactivated HSP70s in the cell according to the presence or absence of stress conditions [15].

Subcellular localization of the HSP70 family members has shown that these genes are located in various parts of the cell, such as the cytoplasm, plastids, endoplasmic reticulum, and mitochondria [16]. Previous studies have indicated the vital roles of the cytosolic HSP70s under stressful [17] and even non-stressful conditions [18]. This highly conserved molecular chaperon has also been reported as a mobile molecule [19] that is able to interact with other stress signals to improve stress tolerance [20]. HSP70s play pivotal roles in response to abiotic, as well as biotic stresses [21,22]. The higher expression of *HSP70* in disease-resistant sunflowers than susceptible genotypes in response to powdery mildew has been reported recently [23]. An up-regulation of *HSP70* was associated with drought tolerance in rice [24], Arabidopsis [25], tobacco [26], sugarcane [27], and chrysanthemum [28]. An overexpression of *CaHSP70*-2 (an *HSP70* gene that belongs to pepper) in Arabidopsis induced the expression of stress-responsive genes and subsequently led to heat tolerance in Arabidopsis [29]. Previous research has indicated that there is an association between HSP70 and ABA (a key stress-responsive phytohormone) in maize under drought and heat stress [30]. Another study predicted that there is a high interaction potential between HSP70 and other phytohormones, such as brassinolide, under stress conditions [31]. Overexpression of an HSP70 gene which belongs to peony (*Paeonia lactiflora* Pall.) in Arabidopsis improved heat tolerance in transgenic Arabidopsis by ameliorating oxidative stress and maintaining cell membrane integrity [32]. According to the diverse functions of HSP70s under different stresses and the crucial role of these molecular chaperones for plant growth and development, this gene family would be a great candidate for improving multiple stresses tolerance [33].

Pumpkin is rich in vitamins, minerals, and antioxidants [34], which has many health benefits, and its seed is a good source for oil extraction [35]. China is the first-ranked country in pumpkin production in the world [36]. This plant species is also one of the most popular rootstocks for cucurbit grafting, which has the ability to improve fruit quality [37], as well as stress tolerance [20,38,39]. In our previous study [40] we identified *HSP70* as the stress-responsive mobile mRNA with a high expression level in response to drought stress in cucumber scion grafted onto pumpkin rootstock. Therefore, we selected this conserved gene family to perform a genome-wide analysis in pumpkin. The genome-wide identification of the *HSP70* gene family has been performed in various species, such as *Nicotiana tabacum* [10], *Solanum tuberosum* [21], *Arabidopsis thaliana* [41], *Glycine max* [42], and rice [17]. However, there is no report of this gene family in pumpkin. Therefore, in this study, we aimed to identify the *HSP70* gene family in pumpkin and analyze their promoter regions, physicochemical characteristics, domains, and evolutionary background. This is the first report on the genome-wide study and expression analysis of *HSP70* in pumpkin rootstock under drought stress. As drought stress takes place in the soil, where the root is, while the related modifications occur in the aerial parts, it is interesting to investigate the collaboration between these two systems. On the other hand, grafted materials are good systems for studying the stress signaling between roots and shoots. Since the signaling molecules are translocated from root to the whole plant body through the vascular system, we used xylem tissue of pumpkin rootstock (in a grafted system with cucumber scion) and checked the expression level of this stress-responsive gene family through grafted materials. It is worth noting that the response of cucumber scion was investigated in our previous study, and a *CmoHSP70* was found as the mobile transcript. Therefore, we studied the response of rootstock in the current study. Our analysis is a helpful source for a deeper understanding of the *CmoHSP70* gene family, which creates a framework for additional investigation of this gene family in pumpkin.

## 2. Results

### 2.1. Identification of HSP70 Gene Family Members in Cucurbita moschata

We could mine 21 *HSP70* genes in *C. moschata* based on the BLASTP method using the domain sequence of Arabidopsis HSP70 as the query. We confirmed the presence of HSP70 domain (PF00012) for all 21 identified *HSP70* genes using Pfam and CDD databases. The detailed information has been provided in the methods section. Then, we named them *CmoHSP70*-1 to *CmoHSP70*-21. The physical and chemical properties of these genes were collected in Table 1. The coding sequence (CDS) and protein length of these genes ranged between 1944 to 9318 bp and 572 to 955 amino acids, respectively. *CmoHSP70*-15 (*CmoCh14G017440*.1) and *CmoHSP70*-21 (*CmoCh09G007070*.1) had the lowest (62.01 kDa) and highest (99.995 kDa) molecular weight (MW). The results of subcellular localization analysis showed that the *CmoHSP70* genes were localized in the cytoplasm, nuclear, or some other organelles, such as endoplasmic reticulum (ER), chloroplast, and mitochondrial. It is worth mentioning that 10 out of 21 *CmoHSP70* genes were predicted to be localized in the cytoplasm. Low isoelectric point (pI) was observed for all the identified genes (5.28 on average) (Table 1). We also searched through a database of nuclear localization signals (NLSdb, https://rostlab.org/services/nlsdb/ (accessed on 11 October 2021)) to find the nuclear signals. The results showed that only two of the genes, *CmoHSP70*-8 (*CmoCh02G009230*.1) and *CmoHSP70*-9 (*CmoCh15G013530*.1), had signals in nuclear (Table S2). 

### 2.2. Evolutionary Relationship, Motif, Gene Structure, and Domain Analysis

**Figure 1:**
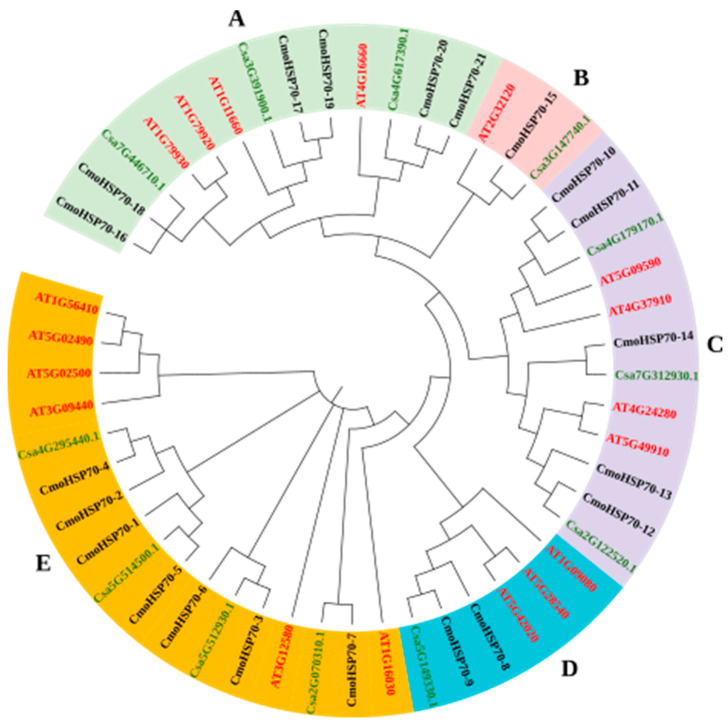
Phylogenetic analysis of HSP70 proteins of pumpkin, cucumber, and Arabidopsis. The HSP70s for pumpkin, Arabidopsis, and cucumber are shown in black, green, and red colors, respectively. The subgroups have been shown in different colors and indicated with the letters A to E.

To investigate the evolutionary relationship of the identified *HSP70s* in *Cucurbita moschata* and other species, we constructed a phylogenetic tree illustrating the evolutionary history of HSP70 proteins among *C. moschata*, *C. sativus*, and *A. thaliana*. Based on the previous studies, the 51 HSP70 sequences were classified into five groups (group A to E) [17,42]. Group B and E contained the least (3 proteins) and most (17 proteins) members, respectively. It is worth mentioning that group A and C had almost similar gene numbers (13 for group A and 12 for group C), and the 6 remaining HSP70 proteins belonged to group D (Figure 1). The phylogenetic tree also indicated a higher similarity between HSP70 sequences of *C. sativus* and *C. moschata* than *A. thaliana*; as cucumber and pumpkin are from the same family, they would be expected to have much more similarity than species from other families (Figure 1). 

**Figure 2:**
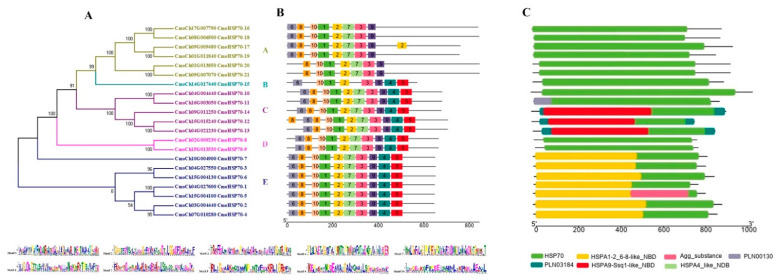
Phylogenetic tree of CmoHSP70 proteins and their motif analysis. (**A**) Phylogenetic tree of CmoHSP70s in pumpkin. The CmoHSP70s were classified in five subgroups, from A to E, based on their similarities to Arabidopsis genes. (**B**) Ten conserved motif proteins of the CmoHSP70s, each small box indicating a motif. All 10 motif logos are shown below the figure. (**C**) Visualization of conserved domains of identified CmoHSP70s using TBtools. Each color represents a specific domain. The corresponding domain names have been shown below the figure.

**Figure 3:**
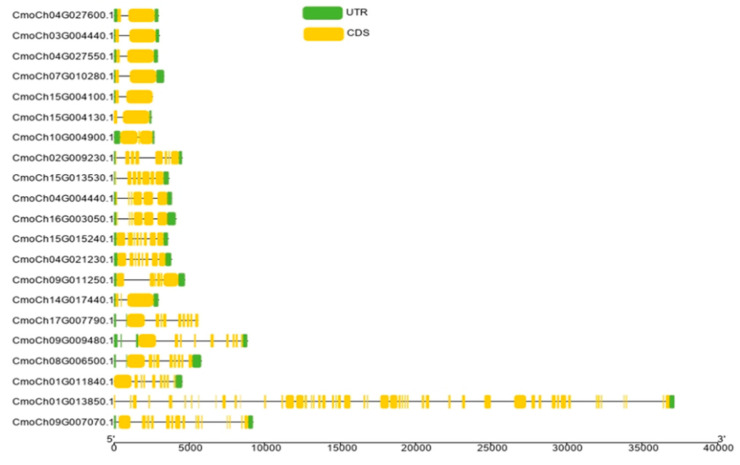
Gene structure analysis of CmoHSP70s in pumpkin. The structures of intron and exon and untranslated regions (UTR) are shown in black line and yellow and green boxes, respectively. The scale is helpful for gene length estimation.

We also established a phylogenetic tree for CmoHSP70 p (Figure 2A) and showed their corresponding motifs in front of each gene. Based on the results of motif analysis, five motifs (motif 10, motif 1, motif 2, motif 3, and motif 9) were in common among all 21 CmoHSP70 proteins. The least number of motifs was found in the proteins belonging to group A. In this group, motif 4 and motif 5 were not identified. Additionally, two of the proteins of this family (CmoCh01G011840 and CmoCh09G007070) did not have motif 6. It is worth mentioning that group B, which contained only one member, lacked motif 7 and motif 8. The subfamily C, D, and E had all 10 motifs (Figure 2B). Domain analysis also revealed that all the CmoHSP70s contained HSP70 and NBD domains (Figure 2C). We furtherly performed multiple sequence alignment using Arabidopsis and pumpkin HSP70 domains. The result indicated a high conservatory of the domains among these species (Figure S1). Gene structure analysis revealed that all genes belonging to subfamily E and B had two to three exons in their CDS sequences. The highest number of exons was observed in group A; *CmoCh01G013850* or *CmoHSP70*-20 with 52 exons had the longest gene length among all *CmoHSP70* genes. The rest of the genes in group A had 9 exons, except *CmoCh09G007070* or *CmoHSP70*-21, which had 15 exons in its structure. The number of exons in groups C and D varied between five to nine. The genes that were distributed in the same group showed a similar number of exons (Figure 3).

### 2.3. Chromosomal Locations and Gene Duplication of the CmoHsp70 Genes in Pumpkin

**Figure 4:**
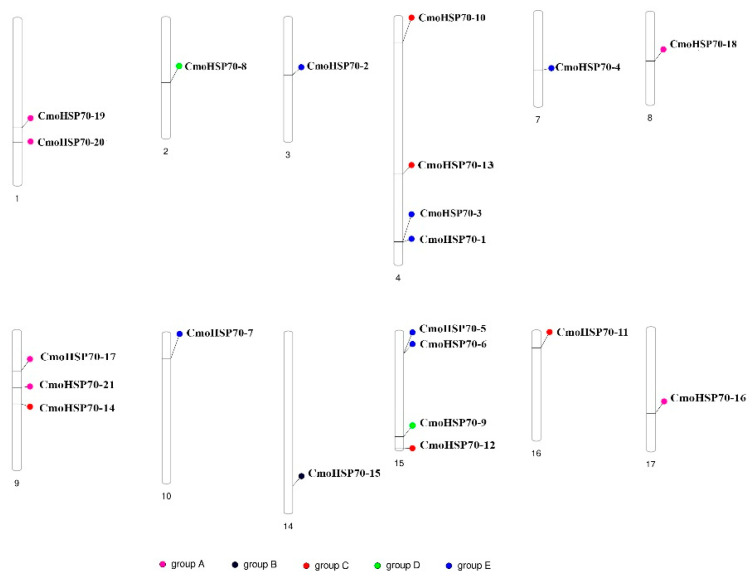
Chromosomal location of the identified *CmoHSP70s* in pumpkin. The genes with the same color indicate that they belong to the same subgroup based on the phylogenetic tree. The chromosome numbers have been shown below them.

Based on the analysis of chromosomal distribution, *CmoHSP70* genes were allocated on 12 out of 20 chromosomes of *C. moschata.* Chromosomes number 4 and 15 carried the most number of *CmoHSP70* genes (each contained four *HSP70* genes), followed by three *CmoHSP70* genes on chromosome number 9. The rest of the chromosomes carried only one *HSP70* gene, except chromosome number 1, which had two genes (Figure 4). 

**Figure 5:**
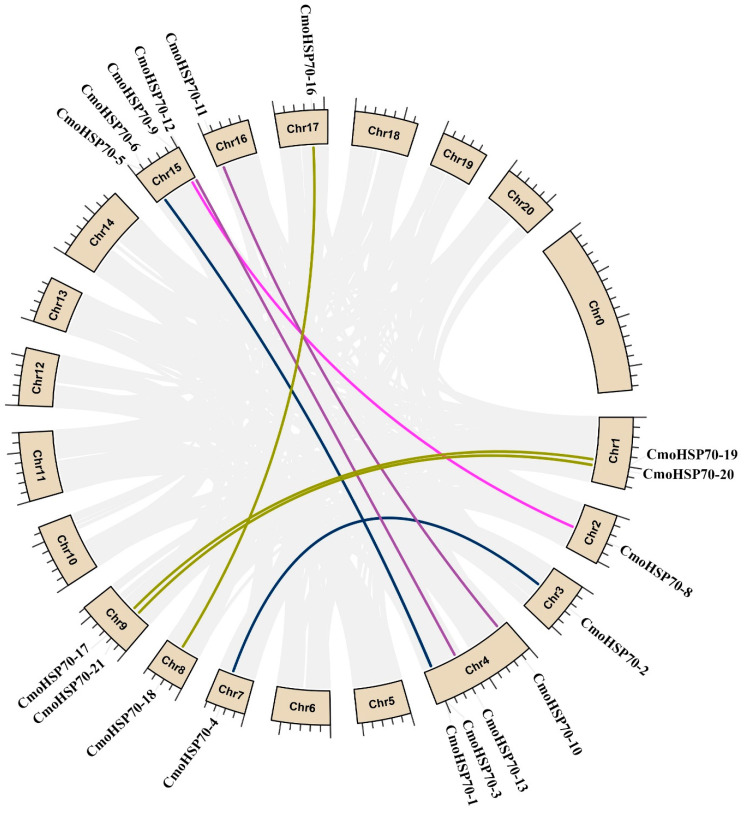
Collinearity analysis of *HSP70* gene family in pumpkin.

**Figure 6:**
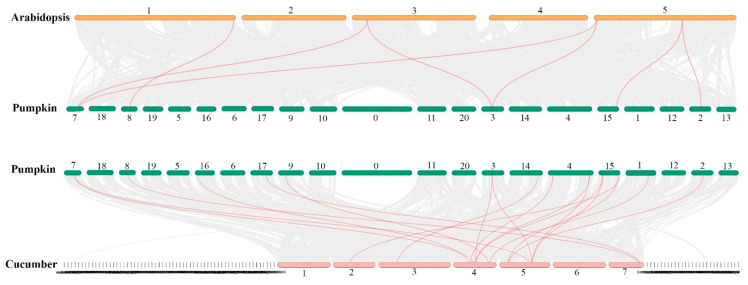
Synteny analysis of *HSP70* family between pumpkin and two other species. The red lines show the *HSP70* orthologous genes between two species, and the gray lines indicate all orthologous genes. The numbers in the figure indicate the chromosome numbers.

We also identified nine pairs of duplicated genes that were located on different chromosomes. Based on the results of gene duplication analysis, all identified paralogous genes had been duplicated as a result of segmental duplication (SD), as they were located on different chromosomes (Figure 5). Ka/Ks (synonymous/non-synonymous) values and duplicated time (T, million years ago (Mya)) were calculated through TBtools and were shown in Table 2. The Ka/Ks values of less than 1 showed the importance of purifying selection in the duplication process. The newest and oldest duplication events occurred around 11 and 101 million years ago (Mya), respectively (Table 2). Dual synteny analysis revealed that there were 7 and 18 *HSP70* orthologs between *C. moschata*/*A. thaliana* and *C. moschata*/*C. sativus*, respectively. It is worth mentioning that five *CmoHSP70* genes were common between the identified orthologous genes in *C. moschata*/*A. thaliana* and *C. moschata*/*C. sativus* (Figure 6 and Tables S3 and S4). 

### 2.4. Promoter Region Analysis and Cis-acting Elements of the CmoHSP70 Genes

**Figure 7:**
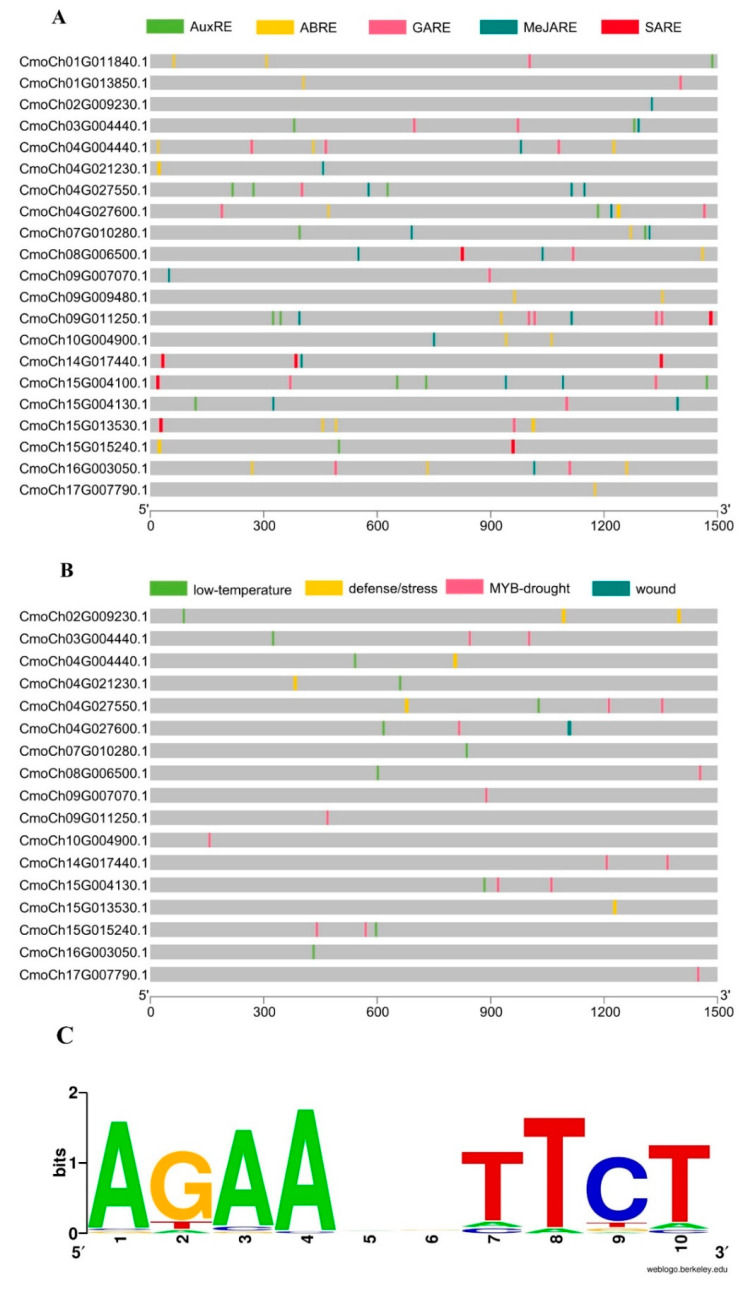
Cis-regulatory elements related to hormones and stress in promoter region of *CmoHSP70* genes. (**A**) The regulatory elements of HSP70 related to hormones and (**B**) indicating the stress-related cis elements. AuxRE (auxin responsive element), ABRE (ABA responsive element), GARE (Gibberellin responsive element), MeJARE (methyl jasmonate responsive element), SARE (salicylic acid responsive element). (**C**) Sequence logo of HSE in the promoter region of CmoHSP70s.

The results of promoter and cis-regulatory analysis of 21 *CmoHSP70* genes revealed that, in total, 88 kinds of cis-regulatory elements were present in the promoter regions of *HSP70* genes of *C. moschata* (Table S5). These elements were categorized into different groups, which have been shown in Table S5. Our analysis showed that 26 out of 88 elements were related to light (light-responsive elements). The elements related to hormones, such as ABA, Ethylene, Auxin, Salicylic acid (SA), Methyl Jasmonate (MeJA), and Gibberellin, were categorized into the second largest group. Other categories, such as stress-related elements, development elements, site binding related elements, and the group of unknown function elements, each contained 10 members. The group of promoter-related elements was the smallest group, with eight cis-regulatory elements. The cis elements related to drought, wounding, low temperature, defense, and MYB-binding site, were among the identified elements related to stress. The cis-regulatory elements related to hormones and stress are shown in Figure 7 and Table S5.

We Furtherly identified the heat shock factor 1 (*HSF1*)-binding motif using PlantPAN3 online database. The promoter sequences of all *CmoHSP70s* were analyzed to recognize the *HSF1*-binding motif, which is also known as HSE, or heat shock element, based on the similarity with the Arabidopsis HSE. The results showed this motif is highly conserved between Arabidopsis and pumpkin. The motif sequences mostly contained two main subunits, including 5′NGAAN3′ and 5′NTTCN3′. The logo of HSE is shown in Figure 7C. Detailed information related to *HSF1*-binding sites for *CmoHSP70s* has been provided in Table S6.

### 2.5. Expression Pattern of HSP70s in Response to Drought Stress

**Figure 8:**
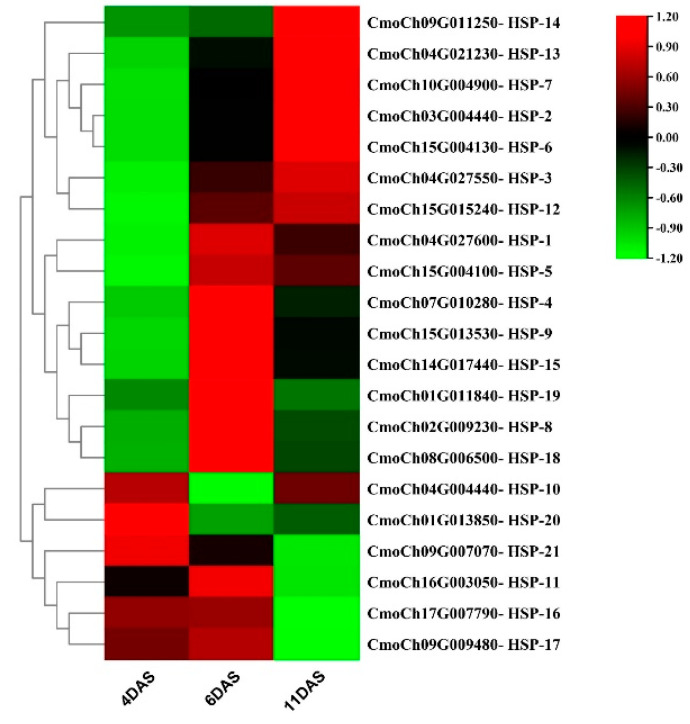
Expression patterns of 21 identified *CmoHSP70* genes in response to drought stress. The samples belonged to the xylem tissues below the graft union of pumpkin rootstock and were collected at 4, 6, and 11 DAS (days after drought stress). Each value is an average of three replications, and each replicate contained three individuals. Green and low colors show low and high relative expression levels, respectively.

Our previous study revealed the presence of the *CmoHSP70* transcript in cucumber scion as a mobile mRNA, which was translocated from pumpkin rootstock with high expression level in response to drought stress. Therefore, in this study we investigated the expression levels of this gene family in response to drought stress. The results indicated that the expression levels of all identified *CmoHSP70s* at 4 days after stress (DAS) were too low. However, by passing the exposure time to drought stress, the expression levels of most of them increased at 6 DAS, except for six of them (*CmoHSP70*-16 to *CmoHSP70*-21). At this time point, *CmoHSP70*-4 (*CmoCh07G010280*) followed by *CmoHSP70*-7 (*CmoCh10G004900*) showed the highest expression level (Figure 8). According to the phylogenetic tree, both of these two genes belonged to group E (Figure 3). The expression patterns of this gene family changed at 11 DAS; while some of them tend to continuously up-regulate, such as *CmoHSP70*-2, 6, 7 (group E), *CmoHSP70*-12, 13, 14 (group C), some others, however, decreased their expression levels under severe drought stress (11 DAS), such as *CmoHSP70*-4, 1, 5 (group E), *CmoHSP70*-8, 9 (group D), *CmoHSP70*-11 (group C), and *CmoHSP70*-15 (group B). It is worth mentioning that the expression levels of all *CmoHSP70s* belonging to group A remained low at all three time points.

## 3. Discussion

HSP70s are classified as chaperones with some pivotal functions, including helping the newly synthesized proteins fold correctly, removing the extra amounts of ROS or damaged proteins under stress conditions, and improving the antioxidant system activity [8]. According to the vital roles of this stress-responsive gene family, the genome-wide study of *HSP70s* has been conducted in model plants, as well as some other plant species, such as tobacco, cotton, maize, potato, and cabbage. However, there was a lack of *HSP70* gene family study in pumpkin as a popular rootstock. Therefore, we extensively performed in silico analysis of this gene family using different bioinformatics tools and investigated its possible role in stress tolerance.

We identified 21 *HSP70* genes in pumpkin, which is equal to the number of *HSP70s* in cotton (*G. arboretum*) [9]. However, soybean[42], cabbage[43], and tobacco[10] have 61, 52, and 61 *HSP70* genes, respectively. It is worth mentioning that potato, maize, and Arabidopsis have almost similar number of *HSP70* genes (18–22 genes) [21,44] to the pumpkin. The different gene numbers of the same family in different species would be because of the size of the genome [42] or evolution diversity [45]. It has been indicated that the *HSP70s* with close phylogenetic relationships are usually located in the same subcellular location and have similar properties or functions [9]. Our results were consistent with this concept, as we could observe more similarity in terms of motifs, the number of introns/exons, and the corresponding cellular compartments in the closely linked subfamilies (Figure 3 and Figure 4). For example, the genes belonging to group E (*HSP70*-1 to *HSP70*-7) were all localized in the cytoplasm and had the same motif distributions (Figure 2). Their gene structures analysis also revealed that these genes all had two exons and one intron, except *HSP70*-7, which had three exons and two introns (Table 1 and Figure 3). These results suggest that the genes from the same group (based on the phylogenetic analysis) might function similarly. Since the gene duplication phenomenon has a crucial effect on the genome evolution of plants [46], we furtherly performed the gene duplication analysis. We could identify nine gene pairs, all of which had been duplicated as a result of segmental duplication (SD), implying the importance of SD phenomenon rather than tandem duplication (TD) in *CmoHSP70* gene expansion (TD) (Figure 5). The significant role of SD rather than TD in *HSP* and *HSF* families expansion has been reported before [47], which is compatible with our findings. It is worth mentioning that a gene duplication process could be considered as segmental once the gene pairs are located on different chromosomes. In contrast, the duplication between genes on the same chromosome is called tandem duplication [48]. The calculation of non-synonymous (Ka) to synonymous (Ks) substitution rate ratio of the gene pairs (paralog genes) is a way to predict the selection method for duplication process [49]. The Ka/Ks values of all *CmoHSP70* paralogs, except one gene pair, were less than 1, implying the involvement of purifying selection (negative selections) in gene duplication (Table 2). It is worth noting that the Ka/Ks ratios greater than 1 show the positive selection (Darwinian selection) [50]. Furtherly, we identified the *CmoHSP70* orthologous genes in other species, such as cucumber and Arabidopsis, and performed synteny analysis. As cucumber and pumpkin are from the same family and are also compatible species for plant grafting, therefore there were more orthologous genes between these two plant species than pumpkin and Arabidopsis (Figure 6 and Tables S3 and S4). These results indicate a closer evolutionary correlation between pumpkin and cucumber rather than pumpkin and Arabidopsis. However, there were five common syntenic genes (*CmoCh08G006500.1*, *CmoCh03G004440.1*, *CmoCh07G010280.1*, *CmoCh02G009230.1*, and *CmoCh15G013530.1*) between these three species, which might indicate the conserved function of these genes across plant species [50]. 

Additionally, the promoter analysis was also carried out, and the cis elements related to hormones and stresses were shown in Figure 7A,B. It has been reported that the cis-regulatory elements contribute to stress responses and regulate the expression of stress-responsive genes [51]. The identified cis elements in our study were mainly related to hormones, environmental stresses, and MYB-binding sites (Table S5). Interestingly, most of the elements were related to ABA (in hormonal class) and drought (in stress group), with 23 and 16 elements, respectively (Table S5). ABA (a well-known stress-responsive phytohormone) and MYB (an important stress-responsive transcription factor) are two key elements for stress tolerance. The regulation of these two stress-responsive elements relies on factors binding to their corresponding cis elements in the promoter region. A recent study has shown that a specific MYB-binding site contributes to drought stress tolerance in wheat [52]. Similarly, another study revealed the importance of the ABRE (ABA-responsive element) for stress signaling, ABA activation, and drought tolerance in Arabidopsis [53]. These results provided more evidence that this gene family plays pivotal roles under stressful conditions. Previous studies have reported the expression level of HSP70s can be regulated through heat shock factor 1 (*HSF1*) [54]. This conserved transcription factor also assists *HSP70* transcripts to translocate from nucleus under stressful conditions [55]. The *HSF1*-binding site, which is known as HSE, was identified in the promoter region of the *CmoHSP70* genes (Table S6), and its logo has also been shown in Figure 7C. The specific sequence of HSE, which can be recognized by HSF1, is NGAAN [56], which is consistent with our results. The interaction between HSP70s and HSF1 has been reported in yeast [57], Arabidopsis [58], and mammalian cells [59].

To investigate the function of the *CmoHSP70s* under stress conditions, we performed the expression analysis of this gene family in the xylem tissue of pumpkin rootstock under drought stress. Dynamic expression analysis of the *CmoHSP70s* showed that some genes were constantly increasing from 4 DAS to 11 DAS, such as CmoHSP70-2, 7, 12, 13, 14, while some others showed a sharp increase at 6 DAS, then decreasing under severe drought stress (*CmoHSP70*-4, 8, 9, 15). There was also another expression pattern in this gene family that can be found in group A. These genes showed continuously low expression levels at all three time points. The induction of most members of this gene family under drought stress indicated the significant roles of these chaperones under stressful conditions (Figure 8). Additionally, the expression patterns of these genes were different in response to drought stress, which implies the diverse roles of the *CmoHSP70s* under stress conditions. These results were consistent with previous studies showing the pivotal functions of *HSP70s* in response to stressful conditions [22,28,60].

## 4. Materials and Methods

### 4.1. CmoHSP70 Sequences Extraction from Cucurbit Database and Collection of their Physicochemical Properties 

A total of 18 *Arabidopsis thaliana HSP70* genes were acquired based on the previous study [41] by searching their gene IDs in TAIR (www.arabidopsis.org/ accessed on 15 October 2021) database. To mine the *C. moschata HSP70* genes, we utilized the *AtHSP70* sequences as queries to withdraw the *CmoHSP70* genes through BLASTP in the *Cucurbit* database (http://cucurbitgenomics.org/ accessed on 15 October 2021). We also found the protein sequence of Arabidopsis HSP70 in NCBI. Then, we performed a sequence search in the Pfam database (http://pfam.xfam.org/ accessed on 15 October 2021) to find its domain sequence. Next, we used the Arabidopsis domain sequence as query to carry out BLASTP against *C. moschata* database. Then, we removed the repetitive genes and applied the E-value < 10^−5^ as criteria for selection. Afterward, we confirmed the presence of the HSP70 domain in all identified *CmoHSP70* genes using SMART (http://smart.embl-heidelberg.de/ accessed on 15 October 2021) and Pfam databases to verify that they belong to the *HSP70* family. Finally, a total of 21 *CmoHSP70* genes were identified as *CmoHSP70* genes in *Cucurbita moschata*. All sequences were provided in Table S1. 

The ExPASy database (https://web.expasy.org/protparam/ accessed on 20 September 2021) was employed to gather the physical and chemical features of *HSP70s*. These properties, including molecular weight (MW), protein length based on the number of amino acids (aa), theoretical isoelectric point (pI), and grand average of hydropathicity (GRAVY), are provided in Table 1. Some other characteristics, such as gene location, strand, and length of coding sequence (bp), were obtained through *Cucurbit* genomic database (http://cucurbitgenomics.org/ accessed on 20 September 2021). Additionally, subcellular localization prediction was performed using online databases, including cello life (http://cello.life.nctu.edu.tw/ accessed on 20 September 2021), and NSLdb (https://rostlab.org/services/nlsdb/ accessed on 20 September 2021). 

### 4.2. Construction of Phylogenetic Tree

The full amino acid sequences of HSP70 proteins belonging to *A. thaliana*, *C. sativus*, and *C. moschata* were downloaded from TAIR (www.arabidopsis.org accessed on 15 October 2021) and *Cucurbit* genomic (http://cucurbitgenomics.org/ accessed on 20 September 2021) databases. The multiple sequence alignment was performed using ClustalW, and the output data were saved in MEGA format. Then, the phylogenetic tree was constructed by MEGA7 software with the method of neighbor-joining (NJ). The Bootstrap method with 1000 replicates was selected as a phylogeny test. Poisson model and complete deletion were chosen in the substitution model and data treatment sections, respectively. iTOL (https://itol.embl.de/ accessed on 15 October 2021) was used to color the phylogenetic tree.

### 4.3. Analysis of Gene Structures, Motifs, and Conserved Domains

TBtools was used to analyze the gene structure of *CmoHSP70* genes using their coding sequences (CDS). To find the conserved motifs of the CmoHSP70 proteins, another online tool was employed, which was Multiple EM for motif elicitation (MEME) (http://meme.nbcr.net/meme3/meme.html/ accessed on 18 October 2021). The maximum number of motifs was selected as 10, and the other parameters were left as default. For domain analysis, we first identified the domain’s type and the position of all HSP70 protein sequences using CDD or NCBI conserved domain database (https://www.ncbi.nlm.nih.gov/Structure/bwrpsb/bwrpsb.cgi/ accessed on 18 October 2021). The results were used for domain visualization using TBtools, a biosequence structure illustrator. Domain sequence alignment of Arabidopsis and pumpkin HSP70 proteins was performed using Mega7 and ClustalW. 

### 4.4. Chromosomal Location, Gene Duplication, and Synteny Analysis

The chromosomal location of the CmoHSP70 genes was shown using an online tool (http://visualization.ritchielab.org/phenograms/plot/ accessed on 18 October 2021). To visualize the paralogous of HSP70 genes, advanced circos plot in TBtools was employed. The required files, including the length of all chromosomes and microsynteny view of gene pairs in pumpkin, were provided using cucurbit database and TBtools. Then, we performed synteny analysis for HSP70 orthologous in pumpkin, cucumber (*Cucumis sativus* L.), and Arabidopsis (*Arabidopsis thaliana*). MCscanX in TBtools was used to conduct dual synteny analysis between pumpkin and the two other species. The information required, such as chromosomal length and whole-genome sequences (GFF3 and fasta format), was downloaded from Cucurbit and phytozome (https://phytozome-next.jgi.doe.gov/ accessed on 18 October 2021) databases. 

### 4.5. Promoter Analysis of the Identified HSP70 Genes of C. moschata

For promoter analysis, we first downloaded 1500 bp of the upstream of identified *CmoHSP70s* from *cucurbit* database. Then, we collected the related information for the promoter regions of these genes through PlantCARE online database (http://bioinformatics.psb.ugent.be/webtools/plantcare/html/ accessed on 20 October 2021). The visualization of cis-regulatory elements related to the hormone and environmental stresses was performed through TBtools. Promoter regions of all CmoHSP70s were also analyzed using PlantPAN3 online tool (http://plantpan.itps.ncku.edu.tw/promoter_multiple.php/ accessed on 20 December 2021). The similarity percentage greater than 0.85 between Arabidopsis and the queries was used to screen the *HSF*-binding sequence or HSEs. Finally, the logo of the HSE sequence was drawn through WebLogo (https://weblogo.berkeley.edu/logo.cgi/ accessed on 20 December 2021) online tool. 

### 4.6. The Analysis of Gene Expression

We used heterografted plants (grafted cucumber onto pumpkin) under drought stress and well-watered conditions, which had been collected and stored in −80 fridge from our previous study. Total RNA was extracted from the xylem tissues below the graft union at 4, 6, and 11 days after drought stress (DAS) using TaKaRa MiniBEST plant RNA extraction kit. cDNA was synthesized by PrimeScript RT reagent Kit with g DNA Eraser (TaKaRa), following the instruction provided in the kit. Then, we performed real-time quantitative PCR (qRT-PCR) by SYBR Premix Ex TaqTM Kit (TaKaRa) in a Bio-Rad iQ1 Real-time PCR system (Bio-Rad). The data were calculated by 2^−ΔΔCt^ method. The final value was calculated as an average of triplicate reactions. Ct value of Cmo-Actin was used to normalize the Ct value of each gene. The list of primers is provided in Table S7.

## 5. Conclusions

In conclusion, genome-wide identification of *CmoHSP70s* in pumpkin revealed that there are 21 genes in this gene family that are unevenly distributed on pumpkin chromosomes. Gene duplication analysis showed that all the *HSP70* paralogous genes in pumpkin are duplicated through SD. The expression analysis of this gene family under drought stress showed that the genes in subfamily E had the highest number of genes, which were located in various organelles, showing the highest expression level in response to drought stress. Interestingly, dynamic expression analysis of these genes at different days after drought stress revealed the different expression patterns, which indicated the diverse function of these genes under stressful conditions. Furtherly, we identified several stress-responsive cis elements in the promoter regions of these genes, which could be the reason for the contribution of *CmoHSP70s* in drought stress tolerance. We believe that these findings will provide valuable information for further investigation of the gene function of this gene family under drought stress.

## Figures and Tables

**Table 1:** Physicochemical properties of identified *CmoHSP70*s in pumpkin.

Transcript ID	Gene Name	Chr.	Location Start-End	CDS (bp)	Protein Length (A.A)	Protein Molecular Weight (kDa)	pI	GRAVY	NO. Intron/Exon	Subcellular Localization ‘Cello Life‘
CmoCh04G027600.1	CmoHSP70-1	4	19963206-19966159	1947	648	71.122	5.17	−0.417	1:2	Cytoplasmic
CmoCh03G004440.1	CmoHSP70-2	3	5028811-5031809	1944	647	70.782	5.13	−0.384	1:2	Cytoplasmic
CmoCh04G027550.1	CmoHSP70-3	4	19938575-19941497	1959	652	71.435	5.1	−0.434	1:2	Cytoplasmic
CmoCh07G010280.1	CmoHSP70-4	7	5088728-5092059	1953	650	71.226	5.16	−0.402	1:2	Cytoplasmic
CmoCh15G004100.1	CmoHSP70-5	15	1867166-1869729	1950	649	71.019	5.16	−0.406	1:2	Cytoplasmic
CmoCh15G004130.1	CmoHSP70-6	15	1874588-1877079	1959	652	71.619	5.11	−0.407	1:2	Cytoplasmic
CmoCh10G004900.1	CmoHSP70-7	10	2190331-2193020	2034	677	71.26	5.15	−0.393	2:3	Cytoplasmic
CmoCh02G009230.1	CmoHSP70-8	2	5674154-5678674	2001	666	73.445	5.07	−0.451	7:8	E.R.
CmoCh15G013530.1	CmoHSP70-9	15	9263256-9266895	1998	665	73.408	5.13	−0.463	6:7	E.R.
CmoCh04G004440.1	CmoHSP70-10	4	2199041-2202895	2043	680	72.994	5.7	−0.309	5:6	Mitochondrial
CmoCh16G003050.1	CmoHSP70-11	16	1399820-1403923	2043	680	73.047	5.7	−0.32	5:6	Mitochondrial
CmoCh15G015240.1	CmoHSP70-12	15	10324523-10328113	2178	725	75.479	5.3	−0.284	8:9	Chloroplast
CmoCh04G021230.1	CmoHSP70-13	4	13928946-13932753	2121	706	75.66	5.26	−0.297	7:8	Chloroplast
CmoCh09G011250.1	CmoHSP70-14	9	6416327-6421038	2130	709	72.927	4.98	−0.298	5:6	Chloroplast
CmoCh14G017440.1	CmoHSP70-15	14	13583561-13586514	1950	572	62.01	5.48	0.027	2:3	Cytoplasmic
CmoCh17G007790.1	CmoHSP70-16	17	7528949-7534538	2523	840	92.562	5.32	−0.425	8:9	Cytoplasmic
CmoCh09G009480.1	CmoHSP70-17	9	4976312-4985160	2283	760	84.941	5.46	−0.41	8:9	Nuclear
CmoCh08G006500.1	CmoHSP70-18	8	4233271-4239067	2532	843	92.659	5.39	−0.42	8:9	Cytoplasmic
CmoCh01G011840.1	CmoHSP70-19	1	9626023-9630560	2277	758	84.845	5.62	−0.411	8:9	Nuclear
CmoCh01G013850.1	CmoHSP70-20	1	10902171-10939329	9318	900	99.903	5.23	−0.488	51:52	Nuclear, ER
CmoCh09G007070.1	CmoHSP70-21	9	3490439-3499658	2868	955	99.995	5.27	−0.481	14:15	ER, Nuclear, Cytoplasmic

**Table 2:** Ka/Ks calculation and estimated divergence time (T) for the duplicated *CmoHSP70* gene pairs.

Gene 1	Gene_2	Ka	Ks	Ka/Ks	Duplication Type	T (MYA) ^1^
*CmoCh17G007790.1*	*CmoCh08G006500.1*	0.02935	0.338926	0.086597	SD ^2^	11.29753381
*CmoCh09G009480.1*	*CmoCh01G011840.1*	0.063151	0.467286	0.135145	SD	15.57619476
*CmoCh01G013850.1*	*CmoCh09G007070.1*	3.304909	3.049809	1.083645	SD	101.6603093
*CmoCh04G004440.1*	*CmoCh16G003050.1*	0.019189	0.364794	0.052602	SD	12.15981475
*CmoCh15G015240.1*	*CmoCh04G021230.1*	0.977111	1.342964	0.727578	SD	44.76545955
*CmoCh02G009230.1*	*CmoCh15G013530.1*	0.00843	0.420521	0.020047	SD	14.01737417
*CmoCh04G027550.1*	*CmoCh15G004130.1*	0.031706	0.633027	0.050087	SD	21.10089572
*CmoCh04G027600.1*	*CmoCh15G004100.1*	0.014152	0.492575	0.028731	SD	16.41915256
*CmoCh03G004440.1*	*CmoCh07G010280.1*	0.01195	0.521627	0.022909	SD	17.38757986

^1^ T = Ks/2λ × 10^−6^ million years ago (Mya), λ = 1.5 × 10^−8^. ^2^ Segmental Duplication.

## Data Availability

The corresponding data have been shown in Supplementary Materials.

## Supplementary Materials

The following supporting information can be downloaded at: https://www.mdpi.com/article/10.3390/ijms23031918/s1.
